# Supplementary material for: Patterns of Intron Gain and Loss in Fungi
Source: PLoS Biol. 2004 Nov 30;2(12):e422. doi: 10.1371/journal.pbio.0020422 (PMC532390; doi:10.1371/journal.pbio.0020422)
Supplement: Table S1 — Also available at http://genes.mit.edu/NielsenEtAl/. (4.3 MB ZIP). [file pbio.0020422.st001.zip › NielsenEtAl/html/668.html]

AN0752.1.NCU05305.1.MG06083.1.FG09748.1


```
 CLUSTAL W (1.82) Multiple Sequence Alignments - Introns Inserted


Sequence 1: NCU05305.1	234 aa
Sequence 2: FG09748.1	253 aa
Sequence 3: AN0752.1	235 aa
Sequence 4: MG06083.1	259 aa
Alignment Length: 270 aa
Number Identitical Residues: 97 aa
Alignment Score (without introns) 4785


MG06083.1 	------MDSNARLAGARKLWRAWRTIHQMLSDR0GYELSEEECNISIERFLADFTDGNGN
NCU05305.1	MEDDS-SPTSA-SKEVSRLWRAWRTVHEMVQDR0GYELAEDEVKISLERFKAEYTNEDGT
FG09748.1 	MDIDH-VPTDAKSKEVVRLWRAWRTVHEMVADR0EYELAEEEVKISLDRFRDEYCNPDGS
AN0752.1  	MDDEYNYSADDAERETIRVWRTWRTVFEMLQDR0GYEVTEEEVKIDLDEFKRKYVGEKGY
          	 . . .  :.     . ::**:***:.:*: **  **::*:* :*.::.*  .: . .* 

MG06083.1 	VD2----QSKLTFSARPSDAMLKLHARIPTPTDPDPAPDCGSIWVEFINEE-KIGVGRTK
NCU05305.1	PN2----RAKLQFSARPSEAMIKKFTAPPTPSNPNPVPECGTIWVEFCTEKGSIGVSVMK
FG09748.1 	IN2INYSRNKLSFSARPSTAMMRKFTPPATAANPDPVADCGPIWIEFLADK-TFGVSQIR
AN0752.1  	PS2----REKLKIQARPTEAMKAKYTPLPTPSNRDPQPDCGTIYIEFCPDDSGIGTKQVR
          	 .     : ** :.***: **   .:  .*.:: :* .:**.*::**  :.. :*.   :

MG06083.1 	QFVEVLLQSGFRDGILVTQGALSSGARRALSATGSGDNKVEAFMLEDLLVNITHHELVPK
NCU05305.1	KFVTHCSDNKFKAGIFITAVPLSAQARKVMSVTSQY-TQVECFLEEDLLVNITHHELVPK
FG09748.1 	AFASYVISNNYKTGIMITHVPLSPAARRSLASVESL-AKIECFLEDDLLVNITHHELVPK
AN0752.1  	TFNHTVDEGNFHTGIFITKTPISPSAVRLLSGIPG--RICEHFQEQDLLVNITRHELVPK
          	 *     .. :: **::*  .:*. * : ::         * *  :*******:******

MG06083.1 	HVMLSRVEKKALLERYRLKETQLPRIQTKDPVARYLGLQRGNVVKIIRHSETAGRYASYR
NCU05305.1	HVLLSKEEKAALLKRYRLKETQLPRILSKDPIARYLGLKRGQVVKIIRVSETAGRYASYR
FG09748.1 	HVLLSREEKIALLKRYRLKETQLPRILQKDPVARYLGLKRGHVVKIIRNSETAGRYASYR
AN0752.1  	HVLLSPEEKSKLLQRYRLKESQLPRIQVSDPVARYLGLRRGQVVKIIRKSETAGRYASYR
          	**:**  **  **:******:*****  .**:******:**:****** ***********

MG06083.1 	L~T0PSVLILQVAISSEKRTPPWLQGGQQSVRSL
NCU05305.1	L~C~V-----------------------------
FG09748.1 	F2Q~GTERASGMRTRASNCK--------------
AN0752.1  	W~V~I-----------------------------
          	
```
